# Intuitive concepts in internal medicine and their occurrence in undergraduate medical students in different semesters

**DOI:** 10.3205/zma001532

**Published:** 2022-02-15

**Authors:** Sigrid Harendza, Christopher Herzog

**Affiliations:** 1 Universitätsklinikum Hamburg-Eppendorf, III. Medizinische Klinik, Hamburg, Germany; 2 Hospital Luneburg, Clinic for Pediatric and Adolescent Medicine, Luneburg, Germany

**Keywords:** misconceptions, internal medicine, intuitive concepts, medical education, undergraduate medical studies, preconcepts

## Abstract

**Background: **Dealing with errors in medical practice is of great importance for patient safety. In the natural sciences, intuitive concepts, so-called misconceptions, are increasingly coming into focus of teaching because they lead to a faulty understanding of contexts and thus to faulty scientific reasoning. In medicine, intuitive concepts still play a subordinate role. However, once intuitive concepts have been memorized, they can become firmly established and, under certain circumstances, lead to diagnostic and treatment errors in medical practice. The aim of this study was to identify potential intuitive concepts in internal medicine and to analyze their occurrence in medical students from different semesters.

**Methods: **Eight internists from different subspecialties were asked about intuitive concepts by means of a structured interview. A total of 17 intuitive concepts were identified. Using these concepts, a multiple-choice test was created with 17 patient cases. For each case, there were four possible answers: the correct answer, an incorrect answer that included the intuitive concept, the answer “both are incorrect”, and the answer “I am not sure”, which is to be understood in the sense of “I do not know whether one of the three answers is correct”. As an online multiple-choice test, these 17 cases were made available to all 2^nd^, 6^th^, and 12^th^ semester students (*N*=1170, *n*=418 from the 2nd semester, *n*=425 from the 6^th^ semester, and *n*=327 from the 12^th^ semester, i.e., the final year) for four weeks in June 2015. The test had to be answered within nine minutes. A mixed logistic regression model was used for evaluation.

**Results: **Of the *N*=317 participating students (*n*=97 from the 2^nd^ semester, *n*=124 from the 6^th^ semester, and *n*=96 from the internship year, overall response rate 27.1%), on average, students from all three groups chose the intuitive concept most often, approximately 40%, although the correct answer increased toward the final year with simultaneously decreasing uncertainty and decreasing feeling of not knowing, respectively. In the final year, compared to the 2^nd^ semester, the intuitive concept was selected significantly more often for two questions (*p*<0.01). For four questions, the intuitive concept was selected significantly less frequently in the final year (*p*<0.01).

**Conclusion:** Intuitive concepts can be identified in internal medicine and do not appear to be significantly reduced in students during the course of their studies. This suggests that this could also be the case for other medical subjects. Therefore, similar studies should be conducted for other medical subjects in order to identify potential sources of error in clinical work. In addition, suitable didactic methods should be developed and tested with which students learn not to succumb to intuitive concepts as far as possible in order to prevent diagnostic or therapeutic errors in later medical practice.

## Zusammenfassung

**Hintergrund: **Der Umgang mit Fehlern ist im ärztlichen Arbeiten für die Patient*innensicherheit von großer Bedeutung. In den naturwissenschaftlichen Fächern rücken intuitive Konzepte, sogenannte Fehlkonzepte, zunehmend in den Fokus der Lehre, da sie zu einem fehlerhaften Verständnis von Zusammenhängen und dadurch zu fehlerhaftem wissenschaftlichem Argumentieren führen. In der Medizin spielen intuitive Konzepte bisher noch eine untergeordnete Rolle. Allerdings können sich einmal eingeprägte intuitive Konzepte nachhaltig festigen und unter Umständen in der ärztlichen Tätigkeit zu Diagnostik- und Behandlungsfehlern führen. Ziel dieser Arbeit war es, potentielle intuitive Konzepte in der Inneren Medizin zu identifizieren und deren Ausprägung bei Medizinstudierenden in verschiedenen Semestern zu analysieren.

**Methoden: **Acht Internistinnen und Internisten verschiedener Teilgebiete wurden mittels eines strukturierten Interviews zu intuitiven Konzepten befragt. Insgesamt konnten 17 intuitive Konzepte identifiziert werden. Mit Hilfe dieser Konzepte wurde ein Multiple-Choice-Bogen mit 17 Patient*innenfällen erstellt. Zu jedem Fall gab es vier Antwortmöglichkeiten: die richtige Antwort, eine falsche Antwort, die das intuitive Konzept beinhaltete, die Antwort „beides ist falsch“ und die Antwort „ich bin mir nicht sicher“, die im Sinne von „ich weiß nicht, ob eine der drei Antworten korrekt ist“ zu verstehen ist. Als Online-Multiple-Choice-Test wurden diese 17 Fälle im Juni 2015 allen Studierenden des 2., 6. und 12. Semesters (*N*=1170, *n*=418 aus dem 2. Semester, *n*=425 aus dem 6. Semester und n=327 aus dem 12. Semester, d.h. dem Praktischen Jahr) für vier Wochen zur Verfügung gestellt. Der Test musste innerhalb von neun Minuten beantwortet werden. Zur Auswertung wurde ein gemischtes logistisches Regressionsmodell verwendet.

**Ergebnisse: **Von den *N*=317 teilnehmenden Studierenden (n=97 aus dem 2. Semester, *n*=124 aus dem 6. Semester und *n*=96 aus dem Praktischen Jahr, Rücklaufquote insgesamt 27,1%) wählten im Mittel Studierende aller drei Gruppen das intuitive Konzept am häufigsten, nämlich zu ca. 40%, obwohl die korrekte Antwort zum PJ hin zunahm bei gleichzeitig abnehmender Unsicherheit bzw. abnehmendem Gefühl des Nicht-Wissens. Im PJ wurde im Vergleich zum 2. Semester bei zwei Fragen das intuitive Konzept signifikant häufiger ausgewählt (*p*<0,01). Bei vier Fragen wurde das intuitive Konzept im PJ signifikant weniger häufig gewählt (*p*<0,01).

**Schlussfolgerung: **Intuitive Konzepte lassen sich in der Inneren Medizin identifizieren und werden bei Studierenden im Laufe des Studiums offenbar nicht wesentlich reduziert. Dies lässt vermuten, dass dies für andere medizinische Fächer auch der Fall sein könnte. Daher sollten ähnliche Untersuchungen auch für weitere medizinische Fächer durchgeführt werden, um potentielle Fehlerquellen für das klinische Arbeiten zu identifizieren. Außerdem sollten geeignete didaktische Methoden entwickelt und überprüft werden, mit denen Studierende lernen, intuitiven Konzepten möglichst nicht zu erliegen, um dadurch diagnostischen oder therapeutischen Fehlern in der späteren ärztlichen Tätigkeit vorzubeugen.

## Introduction

Errors happen more often than expected in the daily clinical routine of physicians and can lead to serious patient danger and death [1]. Dealing with errors openly and developing strategies to avoid them are therefore particularly important in medicine, whereby medicine is often accused of having a rather negative culture of dealing with errors [2], while a positive error culture can serve as a resource for avoiding errors [3]. In the context of quality management in hospitals, there has been a particular focus on the avoidance of technical errors, e.g. the administration of an incorrect intravenous drip, which can be well avoided by the introduction of checklists and leads to higher patient safety [4]. However, not all errors fall into this category, which is why it seems important to be confronted with specific types of errors already during undergraduate education in order to build up an explicit knowledge of errors [5]. Which didactic methods are particularly suitable for this purpose has so far only been researched on an exemplary basis [6]. 

Especially in the natural sciences but also in the humanities, students increasingly describe mental concepts that contradict the content of common scientific concepts [7], [8], [9]. These scientifically untenable notions are referred to as misconceptions or intuitive concepts [10], [11]. They usually stem from simple everyday notions that are often difficult to change despite having been taught in a scientifically correct and the intuitive concept directly contradicting way, because they are deeply embedded in subjective frameworks and have been proven correct in other contexts [12]. Therefore, the term “preconcept” is sometimes used to describe concepts acquired early in non-subject related contexts [13]. A typical intuitive concept from physics, for example, describes the idea of students that force is a cause of motion and that it can be concluded from the presence of motion that a force must be involved. However, this is in direct contradiction to the central statement of Newtonian mechanics and represents an intuitive concept that is retained by students even after successfully passing exams [14]. Therefore, the systematic exploration of intuitive concepts has already led to didactic adaptation of university teaching in some fields [15], [16].

In medicine, too, there are thought processes in the context of clinical reasoning that are based on erroneous concepts and cause so-called cognitive errors, which in turn can lead to incorrect decisions and thus also to treatment errors [17], [18]. Recurring patterns and sequences of decision making required in the diagnostic process may also be the basis for the emergence of intuitive concepts [19]. In particular, medical students and young residents seem to succumb more easily to heuristic misconceptions [20]. Thus, overlaps with intuitive concepts occur here, since misconceptions can have a decisive influence on the scientific decision-making process. For undergraduate medical studies, the presence of intuitive concepts has already been investigated in the subjects of physiology and biochemistry [21], [22]. Furthermore, it has been shown that intuitive concepts were frequently selected by students in a multiple-choice examination despite scientifically based teaching in physiology and biochemistry [23]. Previous teaching methods seem to have limited ability to overcome pre-existing intuitive concepts [24]. In physicians, for example, the presence of intuitive concepts about the causes of fever or the treatment of chronic constipation has been observed [25], [26]. Therefore, the aim of our project was to identify potential intuitive concepts from the field of internal medicine that may also lead to treatment errors under certain circumstances and to investigate their possible occurrence in medical students with different study progress. Our hypothesis was that the choice of intuitive concepts would not decrease with study progress, because the correctly learned content would be further in the past as the education progressed, and intuitive but incorrect answers might therefore predominate for certain questions.

## Project description

### Qualitative identification of potential intuitive concepts in internal medicine

To identify potential intuitive concepts in internal medicine, at first an interview guide was created for discussions with internists. It included the definition of an intuitive concept as well as examples of intuitive concepts from the scientific fields of mathematics [27] and astronomy [28] and from the medical fields of physiology [23] and nephrology [29]. The subsequent questions aimed to generate possible intuitive concepts or theses from the experiences of the physicians interviewed. In addition to the thesis of a potential intuitive concept, the correct scientific explanation of the medical facts was also enquired. A total of seven clinically active male physicians and one female physician from various sub-specialties of internal medicine (diabetology, endocrinology, gastroenterology, infectiology, cardiology, nephrology, pneumology) from the University Medical Center Hamburg-Eppendorf and medical surgeries in Hamburg participated. The interviews were tape-recorded and transcribed verbatim. A total of 17 intuitive concepts could be compiled from the material (see table 1 (Tab. 1)).

#### Development of a case-based multiple-choice test to examine the presence of intuitive concepts in internal medicine

**Figure 1:**
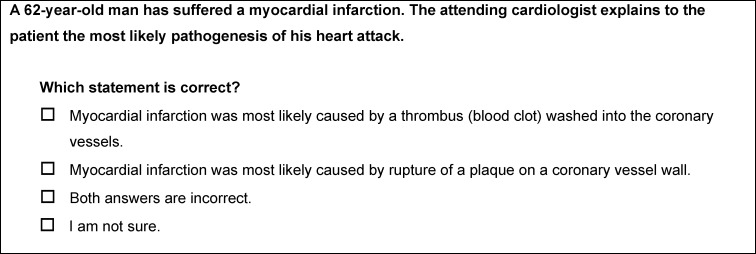
Question of the multiple-choice test on intuitive concept no. 11 (heart attack)

To verify the presence of intuitive concepts in medical students, a case-based multiple-choice test was developed from the 17 intuitive concepts identified. Each question was preceded by a brief case presentation reflecting a typical clinical situation, and there were four response options for each case presentation. These included the correct answer, the incorrect answer that matched the intuitive concept, the answer option “both answers are incorrect”, and the answer choice “I am not sure” in the sense of “I don’t know”. Hence, pressure was to be reduced to have to select one of the answer choices within the guessing probability in case of not knowing the answer. Figure 1 (Fig. 1) exemplary shows the question of the test on intuitive concept no. 11 (heart attack). The complete test with all questions can be found in attachment 1 .

The test was created digitally on an online platform [https://www.umfrageonline.com] and was conducted in multiple-choice style with single choice and no direct provision of the correct answers. Multiple participations were technically prevented by blocking the session browser ID and setting cookies. When clicking on the first page, after a short explanation of the test structure as a novel case-based multiple choice test with four answer options, the socio-demographic data gender, age and current semester of undergraduate medical study were requested. To ensure that the questions could be answered quickly, the test was time-limited to a maximum of nine minutes, of which the participants were informed. The order of the questions was randomized during test creation. Each question was presented on a single page and only after selecting an answer option was it possible to move to the next question. It was not possible to jump back to previous questions.

#### Participants

In June 2015, a total of 1170 undergraduate medical students at the University of Hamburg were invited by e-mail to participate, 418 from the second and 425 from the sixth semester as well as 327 from the final year (PJ). It was possible to participate in two periods of two weeks each. Participation in the multiple-choice test was anonymous and voluntary, and students gave consent to the use of their anonymized data by completing the questionnaire. The study was conducted in accordance with the Declaration of Helsinki and a member of the Ethics Committee of the Hamburg Medical Association had approved this study and confirmed its harmlessness in writing. 

#### Statistical analysis

Statistical analysis of the multiple-choice test was performed using IBM^®^ SPSS^®^ Statistics for Windows, version 23. The probabilities of each student group to choose an answer option were calculated, as well as the corresponding means and standard deviations. A mixed logistic regression model was used for further analysis. Outcome parameters were, on the one hand, the probability of succumbing to the intuitive concept and, on the other hand, the probability of choosing the response option “I am not sure” as a measure of uncertainty in the sense of not knowing. The variables used as predictors were the level of education (current semester) and the individual questions of the test. The interaction was tested using likelihood ratio. Gender and age were considered as confounders. Due to the multiple measurements per participant, these had to be modeled as clusters in the logistic regression. The adjusted probabilities of the individual answer options of the multiple-choice test for the subgroups and their pairwise comparison with the corresponding 95% confidence intervals are reported.

## Results

A total of 317 of 1170 invited students participated in the online multiple choice test (response rate 27.1%), of which 97 were from semester 2, 124 from semester 6, and 96 from the final year (see table 2 (Tab. 2)). The mean age of all participants was 24.5±4.2 year and 61.2% of participants were female. The proportion of correct answers was greater for final-year students (39.1%) compared with 2^nd^ semester (22.6%) and 6^th^ semester (32.1%) students, and uncertainty or feeling of not knowing the answer was lowest (final year: 5.7%, 2^nd^ semester: 32.0%, 6^th^ semester: 16.8%). However, the selection of the intuitive concept was approximately 40% overall and represented in all three groups the largest percentage of all responses. Attachment 2 shows the percentages of responses related to each question of the multiple-choice test. The numbers of answers per individual question can be found in attachment 3 .

**Figure 2:**
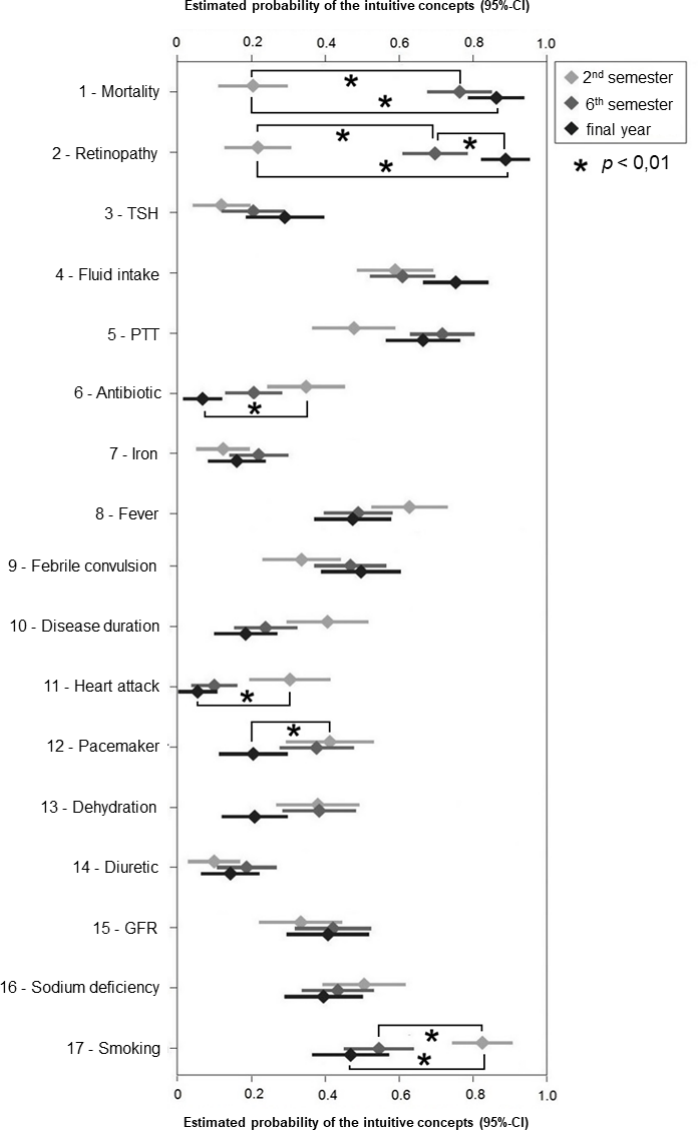
Estimated probabilities of intuitive concepts per question and semester

Differences emerged for the individual subgroups “2^nd^ semester”, “6^th^ semester”, and “final year” in terms of the calculated probabilities of succumbing to the intuitive concept (see figure 2 (Fig. 2)). For 9 of the 17 intuitive concepts examined, there was an increase in the probability of selecting the intuitive concept among final-year students compared with 2^nd^ semester students. For the choice of intuitive concepts 1 (“mortality”) and 2 (“retinopathy”), the probability was even significantly (p=0.01) increased for final-year students compared to both 2^nd^ semester and 6^th^ semester students. Also significantly (p=0.01) greater for these two questions was the probability of succumbing to the intuitive concept for 6^th^ semester students compared to 2^nd^ semester students. For intuitive concepts 6 (“antibiotic”), 11 (“heart attack”), 12 (“pacemaker”), and 17 (“smoking”), the probability of choosing the intuitive concept was significantly (p=0.01) lower in final-year studentss compared to 2^nd^ semester students. There was no correlation with age and gender.

**Figure 3:**
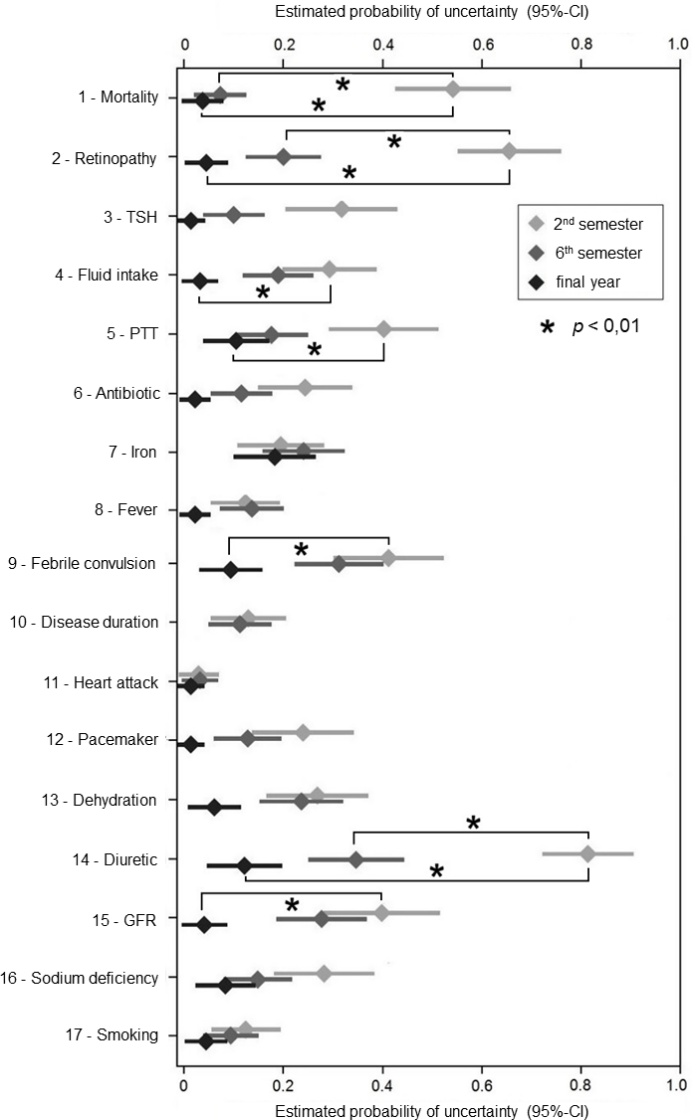
Estimated probabilities of uncertainty or feeling not to know the answer, respectively, per question and semester

Uncertainty in selecting an answer or the feeling of not knowing the answer, respectively (see figure 3 (Fig. 3)), was shown to be significantly (p=0.01) less in final-year students compared to 2^nd^ semester students for intuitive concepts 1 (“mortality”), 2 (“retinopathy”), 4 (“fluid intake”), 5 (“PTT”), 9 (“febrile seizure”), 14 (“diuretic”), and 15 (“GFR”), although at the same time the probability of succumbing to the intuitive concept increased (see figure 2 (Fig. 2)). Intuitive concepts 1 (“mortality”), 2 (“retinopathy”), and 14 (“diuretic”) were also significantly less uncertain for 6^th^ semester students compared with 2^nd^ semester students.

## Discussion

By interviewing internal medicine residents, we identified 17 intuitive concepts from the field of internal medicine. Although in our multiple-choice test, which included questions on these concepts, the selection of the correct answer increased with increasing semester among the medical students and the uncertainty in answering the questions or the feeling of not knowing the answer decreased, the intuitive concept was chosen as the most frequent answer by students of all semesters. In a study on causes of worsening acne vulgaris, it was also shown that intuitive concepts common in the general population were still highly prevalent among medical students in their final year of study [30]. The consistent percentage per semester of intuitive concept selection for internal medicine topics in our study confirmed studies in medical physiology that existing intuitive concepts appeared to have little to no ability to be overcome by previous teaching methods [24], [31]. Another study of biomedical science students, using a multilayered approach to study intuitive concepts in cardiovascular physiology, could demonstrate that in nearly one-third of the cases the selection of the intuitive concept was paired with high confidence of having answered the question correctly [32]. Unlike in our multiple-choice test, here the degree of uncertainty about the selected answer of the concept should be indicated, whereas in our test not knowing was a separate answer option.

Since intuitive concepts on medical aspects impair clinical reasoning and this ignorance may possibly lead to cognitive thinking errors in medicine [33], [34] and thus also to treatment errors, it seems necessary, on the one hand, to identify possible intuitive concepts in the different medical disciplines, as in our study for internal medicine. On the other hand, didactic methods need to be tested that help students to permanently overcome intuitive concepts in medicine. So far, it has been shown that teaching on conceptual change was more helpful for this purpose [35] than supplementary facts or feedback [36], [37]. For some of the intuitive concepts we identified, e.g., “heart attack” and “antibiotic”, this may perhaps have been the reason for a reduction in intuitive concept use among students in higher semesters. However, it was also shown that knowledge in evidence-based medicine, which was supposed to counteract medical performance according to intuitive concepts with scientific justifications, deteriorated in students during the final year, whereas this was not the case for knowledge on clinical cases requiring urgent treatment [38]. This suggests that in addition to conceptual changes in scientific thinking, working with mnemonic bridges might also be useful, as has been successfully shown for the use of antibiotics in veterinary medicine [39] or for generating a larger number of differential diagnoses in medicine [40]. However, this assumes that possible intuitive concepts are known. If this is the case, such techniques could be complementarily helpful to avoid having to remember the correct scientific background during fast clinical decisions, as simulated by the time limit in our multiple-choice test, and then mistakenly succumbing to the intuitive concept after all.

In our study, we were able to show for the first time that intuitive concepts exist in the various fields of internal medicine. However, a weakness of the project is that not all areas of internal medicine were investigated and that it was not a systematic survey, so that the number of intuitive concepts identified could be incomplete. In addition, in the design of the multiple-choice test, the item “I don’t know” should have been chosen instead the item “I am not sure”, since the question was about knowledge of a concept and not about the feeling of uncertainty in, for example, medical-diagnostic decisions [41]. Furthermore, it was not taken into account that despite the four possible answers there may have been a certain guessing probability, since the distractor “both are wrong” is rather weak and “I don’t know” is not a real distractor. This weakness could have been reduced by introducing two other distractors. Alternatively, an open answer with subsequent coding by two independent raters would have been conceivable. In addition, an interferential statistical analysis of the correct answer and the influence of the level of education would have been interesting for this multiple-choice test. The survey of students used a convenience sample, which limits the interpretation of our findings. In addition, the response rate was only just under one third of the respondents, which could have led to a bias, as possibly only particularly interested or particularly good students could have participated in the study. Furthermore, since this is not a longitudinal study, no conclusions can be drawn about curricular aspects or didactic teaching methods. Nevertheless, our study offers first insights that intuitive concepts also exist in internal medicine and that these do not seem to change significantly proportionally with study progress in medical students, which is in line with the initial hypothesis. In some cases, they even increase. As in the natural sciences, intuitive concepts learned from other life contexts [13] seem to overwrite scientific explanations learned in medical school [14], especially when the distance to these curricular contents is large, time pressure exists, or the scientific concept is no longer constantly needed in everyday life. These findings provide initial starting points to focus on correctly learning essential aspects in internal medicine that may lead to later treatment errors due to intuitive concepts, so that students can build error knowledge [5]. The detection of intuitive concepts could also be suitable for other subjects at medical school in order to subsequently develop didactic approaches, e.g. using mnemonic bridges [40], to support students as future physicians not to succumb to intuitive concepts when they encounter them in clinical routine and under time pressure.

## Conclusion

Our study was able to show that diverse intuitive concepts exist in internal medicine and that these were most frequently selected by medical students in a multiple-choice examination regardless of semester, although uncertainty or the feeling of not knowing the answer decreased with an increasing number of semesters. It is reasonable to assume that intuitive concepts exist for other medical specialties that may lead to diagnostic or therapeutic errors. Therefore, further studies should be conducted for both internal medicine and other medical specialties to identify intuitive concepts. Intervention studies are also needed to support students with didactic concepts to learn strategies to avoid succumbing to intuitive concepts in later professional life and to make incorrect diagnoses or provide wrong treatments.

## Acknowledgement

We thank all involved physicians and medical students for their participation.

## Competing interests

The authors declare that they have no competing interests. 

## Supplementary Material

Individual questions with the respective intuitive concept and the correct answer

Percentage of answers per individual question

Number of answers per individual question

## Figures and Tables

**Table 1:**
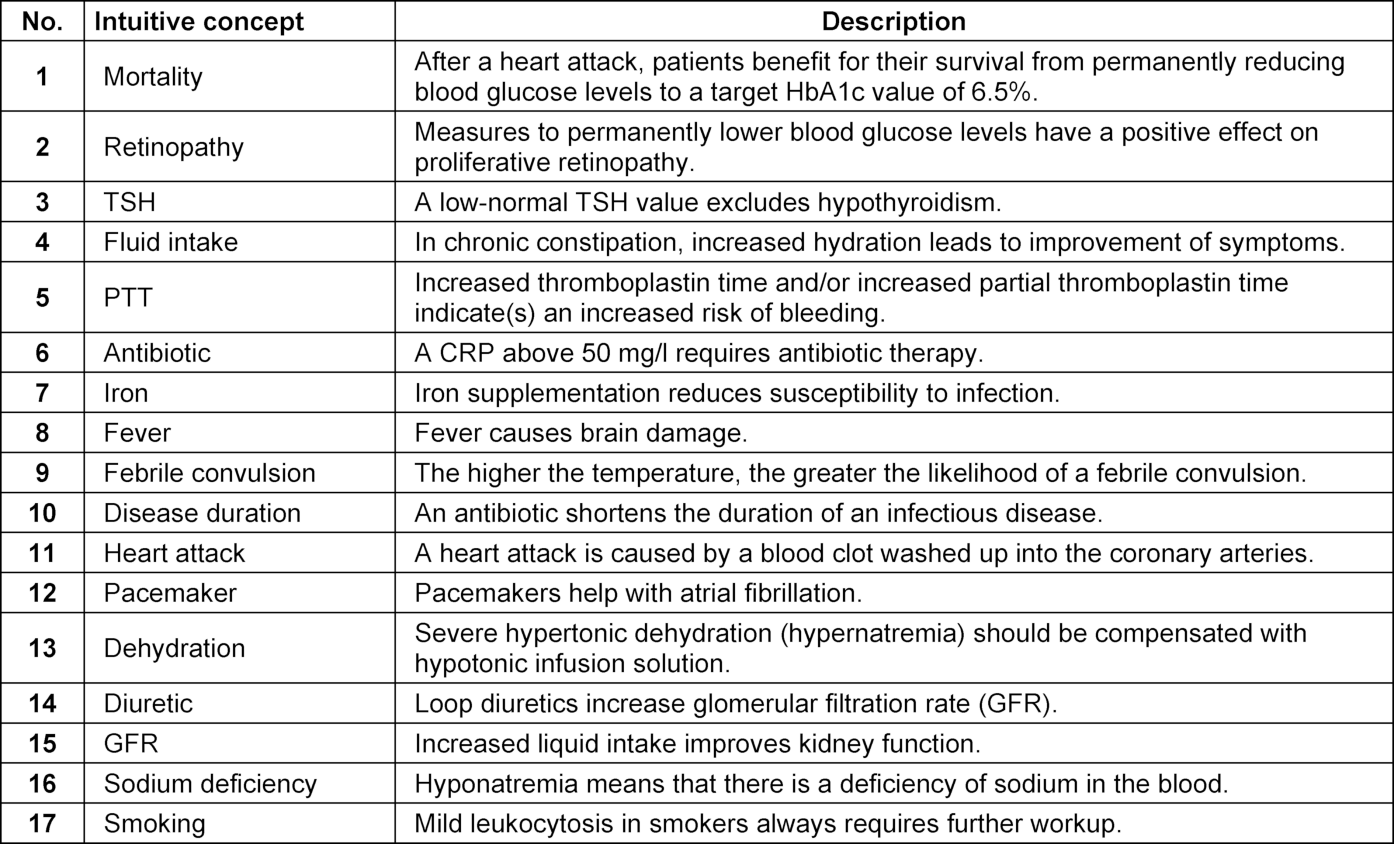
Intuitive concepts identified in internal medicine

**Table 2:**
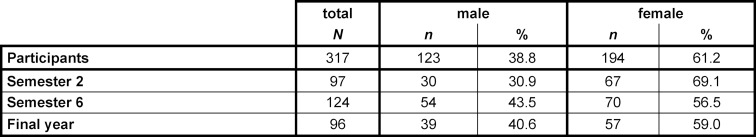
Sociodemographic data of the participating students

## References

[1] Krohn LT, Corrigan JM, Donaldson MS (1999). To err is human: building a safer health system.

[2] Wilkesmann M, Steden S, Wilkesmann M, Steden S (2019). Nichtwissen als Problem - „Ärzte machen keine Fehler“. Nichtwissen stört mich (nicht). Zum Umgang mit Nichtwissen in Medizin und Pflege.

[3] Haller U, Welti S, Haenggi D, Fink D (2005). Von der Schuldfrage zur Fehlerkultur in der Medizin. Gynäkol Geburtshilfliche Rundsch.

[4] Gawande A (2009). The checklist manifesto. How to get things right.

[5] Oser F, Hascher T, Spychiger M, Althoff W (1999). Lernen aus Fehlern. Zur Psychologie des „negativen“ Wissens. Fehlerwelten: Vom Fehlermachen und Lernen aus Fehlern.

[6] Sibbald M, Sherbino J, Ilgen JS, Zwaan L, Blissett S, Monteiro S, Norman G (2019). Debiasing versus knowledge retrieval checklists to reduce diagnostic error in ECG interpretation. Adv Health Sci Educ Theory Pract.

[7] Riegler P (2014). Schwellenkonzepte, Konzeptwandel und die Krise der Mathematikausbildung. Z Hochschulentwick.

[8] Middendorf J, Pace D (2004). Decoding the disciplines: a model for helping students learn disciplinary ways of thinking. New Dir Teaching Learn.

[9] Meyer JH, Land R, Rust C (2003). Threshold concepts and troublesome knowledge: linkages to ways of thinking and practicing within the disciplines. Improving student learning: improving student learning theory and practice - ten years on.

[10] Taylor AK, Kowalski P (2004). Naïve psychological science: the prevalence, strength, and sources of misconceptions. Psychol Rec.

[11] Chi MT, Roscoe RD, Slotta JD, Roy M, Chase CC (2012). Misconceived causal explanations for emergent processes. Cogn Sci.

[12] Vosniadou S (1994). Capturing and modeling the process of conceptual change. Learn Instruct.

[13] Vosniadou S, Schnotz W, Vosniadou S, Carretero M (1999). Conceptual change research: state of the art and future directions. New perspectives on conceptual change.

[14] Hake RR (1998). Interactive-engagement versus traditional methods: A six-thousand-student survey of mechanics test data for introductory physics courses. Am J Physics.

[15] Redish EF, Burciaga JR (2003). Teaching physics with the physics suite. Am J Physics.

[16] Arnon I, Cottrill J, Dubinsky E, Oktaç A, Fuentes SR, Trigueros M, Weller, K (2014). The teaching of mathematics using APOS theory.

[17] Croskerry P (2003). The importance of cognitive errors in diagnosis and strategies to minimize them. Acad Med.

[18] Klinge A, Müller J, Harendza S (2019). Wie Denkfehler die ärztliche Diagnose beeinflussen. Hamb Arztebl.

[19] Elstein AS (1999). Heuristics and biases: selected errors in clinical reasoning. Acad Med.

[20] Rylander M, Guerrasio J (2016). Heuristic errors in clinical reasoning. Clin Teach.

[21] Michael JA (1998). Students' misconceptions about perceived physiological responses. Am J Physiol.

[22] Oliveira G, Sousa C, Poian A, Luz M (2003). Students' misconception about energy-yielding metabolism: glucose as the sole metabolic fuel. Adv Physiol Educ.

[23] Morton JP, Doran DA, MacLaren DP (2008). Common student misconceptions in exercise physiology and biochemistry. Adv Physiol Educ.

[24] Palizvan MR, Nejad MR, Jand A, Rafeie M (2013). Cardiovascular physiology misconceptions and the potential of cardiovascular physiology teaching to alleviate these. Med Teach.

[25] Demir F, Sekreter O (2012). Knowledge, attitudes and misconceptions of primary care physicians regarding fever in children: a cross sectional study. Ital J Pediatr.

[26] Müller-Lissner SA, Kamm MA, Scarpignato C, Wald A (2005). Myths and misconceptions about chronic constipation. Am J Gastroenterol.

[27] Gürbüz R, Birgin O (2012). The effect of computer-assisted teaching on remedying misconceptions: The case of the subject "probability". Comput Educ.

[28] Kalkan H, Kiroglu K (2007). Science and nonscience students' ideas about basic astronomy concepts in preservice training for elementary school teachers. Astron Educ Rev.

[29] Wenzel UO, Hebert LA, Stahl RA, Krenz I (2006). My doctor said I should drink a lot! Recommendations for fluid intake in patients with chronic kidney disease. Clin J Am Soc Nephrol.

[30] Green J, Sinclair RD (2001). Perceptions of acne vulgaris in final year medical student written examination answers. Australas J Dermatol.

[31] Bordes SJ, Gandhi J, Bauer B, Protas M, Solomon N, Bogdan L, Brummund D, Bass B, Clunes M, Murray IVJ (2020). Using lectures to identify student misconceptions: a study on the paradoxical effects of hyperkalemia on vascular smooth muscle. Adv Physiol Educ.

[32] Versteeg M, Wijnen-Meijer M, Steendijk P (2019). Informing the uninformed: a multitier approach to uncover students' misconceptions on cardiovascular physiology. Adv Physiol Edu.

[33] Woods NN (2007). Science is fundamental: the role of biomedical knowledge in clinical reasoning. Med Educ.

[34] Berner ES, Graber ML (2008). Overconfidence as a cause of diagnostic error in medicine. Am J Med.

[35] Hewson MG, Hewson PW (1983). Effect of instruction using students' prior knowledge and conceptual change strategies on science learning. J Res Sci Teach.

[36] Ecker UK, Lewandowsky S, Tang DT (2010). Explicit warnings reduce but do not eliminate the continued influence of misinformation. Mem Cognit.

[37] Archer JC (2010). State of the science in health professional education: effective feedback. Med Educ.

[38] Heidemann LA, Keilin CA, Santen SA, Fitzgerald JT, Zaidi NL, Whitman L, Jones EK, Lypson ML, Morgan HK (2019). Does performance on evidence-based medicine and urgent clinical scenarios assessments deteriorate during the fourth year of medical school? Findings from one institution. Acad Med.

[39] Cole SD, Elliott ER, Rankin SC (2021). SODAPOP: A Metacognitive Mnemonic Framework to Teach Antimicrobial Selection. J Vet Med Educ.

[40] Leeds FS, Atwa KM, Cook AM, Conway KA, Crawford TN (2020). Teaching heuristics and mnemonics to improve generation of differential diagnoses. Med Educ Online.

[41] Wübken M, Oswald J, Schneider A (2013). Umgang mit diagnostischer Unsicherheit in der Hausarztpraxis. Z Evid Fortbild Qual Gesundhwes.

