# Supplementary material for: Intuitive concepts in internal medicine and their occurrence in undergraduate medical students in different semesters
Source: GMS J Med Educ. 2022 Feb 15;39(1):Doc11. doi: 10.3205/zma001532 (PMC8953187; doi:10.3205/zma001532)
Supplement: Individual questions with the respective intuitive concept and the correct answer [file JME-39-11-s-001.pdf]

Attachment 1: Individual questions with the respective intuitive concept and the correct answer

Question 1: Mortality

A 69-year-old man has suffered a first myocardial infarction. The secondary disease is type II diabetes mellitus, previously treated with metformin (oral antidiabetic) (HbA1c currently 9%). (normal values HbA1c 4.4 – 6.0 %).

- ☐ Target HbA1c values of 6.5% and 7%, respectively, are more likely to result in lower mortality in patients with diabetes mellitus and post-myocardial infarction status.
- ☐ Target HbA1c values of 6.5% and 7%, respectively, are more likely to result in higher mortality in patients with diabetes mellitus and post-myocardial infarction status.
- ☐ Both answers are incorrect.
- ☐ I am not sure.

Question 2: Retinopathy

A 75-year-old man suffers from difficult-to-control type II diabetes mellitus. The patient has also a history of diabetic retinopathy. The current HbA1c value is 8.9% (normal values HbA1c 4.4 – 6.0%).

- ☐ Lowering blood glucose levels (target HbA1c 6.5 – 7%) reduces progression of proliferative retinopathy.
- ☐ Lowering blood glucose (target HbA1c 6.5 – 7%) no longer affects progression of proliferative retinopathy.
- ☐ Both answers are incorrect.
- ☐ I am not sure.

Question 3: TSH

A 34-year-old woman presents to her general praticioner’s office with weight gain, constipation, and fatigue. Among other things, the hormone TSH is measured by the laboratory. Result: 0.5 mU/l (normal values 0.3 – 4.0 mU/l).

- ☐ Hypothyroidism has been ruled out.
- ☐ Hypothyroidism is not ruled out.
- ☐ Both answers are incorrect.
- ☐ I am not sure.

Question 4: Fluid intake

A 54-year-old woman suffers from chronic constipation. The woman estimates her daily drinking quantity as approx. 1.2 L.

- ☐ A significantly higher fluid intake (approx. 3 L per day) can improve the symptoms.
- ☐ A significantly higher fluid intake (approx. 3 L per day) cannot improve the symptoms.
- ☐ Both answers are incorrect.
- ☐ I am not sure.

Question 5: PTT

A 24-year-old man is scheduled for elective knee surgery. The laboratory examination reveals an elevated partial thromboplastin time (PTT). All other laboratory parameters were unremarkable.

- ☐ The patient is likely to be at increased risk of bleeding.
- ☐ The patient is not expected to be at increased risk of bleeding.
- ☐ Both answers are incorrect.
- ☐ I am not sure.

Question 6: Antibiotic

A 24-year-old man has been suffering from vomiting and diarrhea since eating a bratwurst the previous day and presents to the general practitioner’s office. Laboratory chemistry reveals a CRP (inflammation) value of 62 mg/l (normal: below 5 mg/l).

- ☐ CRP above 50 mg/l requires antibiotic therapy.
- ☐ CRP above 50 mg/l does not require antibiotic therapy.
- ☐ Both answers are incorrect.
- ☐ I am not sure.

Question 7: Iron

A 32-year-old woman has frequent infections and presents to her general practitioner with the question of whether she should take iron tablets to prevent further infections because her blood iron level is very low.

- ☐ In this case, increased oral iron intake reduces susceptibility to infection.
- ☐ In this case, increased oral iron intake does not reduce susceptibility to infection.
- ☐ Both answers are incorrect.
- ☐ I am not sure.

Question 8: Fever

A fever of 40.2°C is measured in a 36-year-old woman in the course of a severe infection.

- ☐ The patient must receive antipyretic medication in addition to antibiotic therapy because fever above 39°C causes damage to brain tissue.
- ☐ The patient does not need to receive antipyretic medication in addition to antibiotic therapy because fever above 39°C does not cause damage to brain tissue.
- ☐ Both answers are incorrect.
- ☐ I am not sure.

Question 9: Febrile convulsion

The parents of a two-month-old child would like to be informed by their primary care physician regarding febrile seizures.

- ☐ The higher the fever, the higher the likelihood of febrile seizure.
- ☐ The level of fever has no significance for the occurrence of febrile convulsions.
- ☐ Both answers are incorrect.
- ☐ I am not sure.

Question 10: Disease duration

A 21-year-old woman suffers from bronchitis with fever (up to 39.1°C), headache and pain in the limbs, cough with scanty, viscous sputum and a sore throat. She wants to be quickly healthy again and free of fever due to upcoming exams.

- ☐ In addition to ample fluid intake for good secretolysis, an antibiotic should be recommended for the aforementioned symptoms, as it is very likely to significantly shorten the duration of the disease.
- ☐ In addition to ample hydration for good secretolysis, antibiotics should not be recommended for the aforementioned symptoms, as they are very unlikely to significantly shorten the duration of the disease.
- ☐ Both answers are incorrect.
- ☐ I am not sure.

Question 11: Heart attack

A 62-year-old man has suffered a myocardial infarction. The attending cardiologist explains to the patient the most likely pathogenesis of his myocardial infarction.

- ☐ Myocardial infarction was most likely caused by a thrombus (blood clot) washed into the coronary vessels.
- ☐ Myocardial infarction was most likely caused by rupture of a plaque on a coronary vessel wall.
- ☐ Both answers are incorrect.
- ☐ I am not sure.

Question 12: Pacemaker

A 63-year-old woman is diagnosed with atrial fibrillation. She suffers from palpitations, irregular pulse and dizziness.

- ☐ Implantation of a pacemaker is indicated because the patient is symptomatic due to atrial fibrillation.
- ☐ Implantation of a pacemaker is not indicated although the patient is symptomatic due to atrial fibrillation.
- ☐ Both answers are incorrect.
- ☐ I am not sure.

Question 13: Dehydration

A 78-year-old woman is admitted to the emergency department with severe hypertonic dehydration (hypernatremia).

- ☐ Hypertonic dehydration should be compensated by hypotonic saline.
- ☐ Hypertonic dehydration should be compensated by isotonic saline.
- ☐ Both answers are incorrect.
- ☐ I am not sure.

Question 14: Diuretic

A 72-year-old man is admitted for elective surgery. He has chronic renal failure with a serum creatinine of 2.8 mg/dl (normal value below 1.2 mg/dl). The anesthesiologist wants the serum creatinine to be lowered preoperatively.

- ☐ Creatinine can be lowered in this patient with a loop diuretic.
- ☐ Creatinine cannot be lowered with a loop diuretic in this patient.
- ☐ Both answers are incorrect.
- ☐ I am not sure.

Question 15: GFR

A 72-year-old man is found to have a decreased glomerular filtration rate (GFR) of 65 ml/min with otherwise normal laboratory parameters. His daily fluid intake is approx. 1.3 L.

- ☐ Increasing the fluid intake leads to an improvement in GFR.
- ☐ Increasing the fluid intake does not improve GFR.
- ☐ Both answers are incorrect.
- ☐ I am not sure.

Question 16: Sodium deficiency

An 80-year-old woman is admitted to the central emergency room after a fall in a nursing facility. The laboratory reveals a sodium value of 118 mmol/l (normal sodium values: 135 – 148 mmol/l).

- ☐ In the serum, the patient is deficient in sodium relative to water.
- ☐ In the serum, the patient has excess water in relation to the amount of sodium.
- ☐ Both answers are incorrect.
- ☐ I am not sure.

Question 17: Smoking

A 42-year-old man presents to his general practitioner's office for a check-up. He has hypercholesterolemia, exercises regularly, and is a smoker (20 PY). Laboratory chemistry reveals a leukocytosis of 12,000/µl. Normal values (3,800 – 10,500/µl).

- ☐ In this case, mild leukocytosis requires further workup.
- ☐ In this case, mild leukocytosis does not require further workup.
- ☐ Both answers are incorrect.
- ☐ I am not sure.
