# Supplementary material for: Intuitive concepts in internal medicine and their occurrence in undergraduate medical students in different semesters
Source: GMS J Med Educ. 2022 Feb 15;39(1):Doc11. doi: 10.3205/zma001532 (PMC8953187; doi:10.3205/zma001532)
Supplement: Percentage of answers per individual question [file JME-39-11-s-002.pdf]

Attachment 2: Percentage of answers per individual question

Question 1: Mortality (*n* = 244)

	Correct answer	Intuitive concept	Both are incorrect	Uncertainty
**Semester 2**	15,3 %	20,8 %	9,7 %	54,2 %
**Semester 6**	10,8 %	76,3 %	5,4 %	7,5 %
**Final year**	5,1 %	86,1 %	5,1 %	3,7 %
**Total**	10.2 %	63,1 %	6,6 %	20,1 %

Question 2: Retinopathy (*n* = 274)

	Correct answer	Intuitive concept	Both are incorrect	Uncertainty
**Semester 2**	12,4 %	22,2 %	0,0 %	65,4 %
**Semester 6**	7,6 %	69,5 %	2,9 %	20,0 %
**Final year**	4,5 %	88,7 %	2,3 %	4,5 %
**Total**	8,0 %	61,7 %	1,8 %	28,5 %

Question 3: TSH (*n* = 224)

	Correct answer	Intuitive concept	Both are incorrect	Uncertainty
**Semester 2**	54,6 %	12,1 %	1,5 %	31,8 %
**Semester 6**	66,7 %	20,7 %	2,3 %	10,3 %
**Final year**	66,2 %	29,6 %	2,8 %	1,4 %
**Total**	63,0 %	21,0 %	2,2 %	13,8 %

Attachment 2 to Harendza S, Herzog C. *Intuitive concepts in internal medicine and their expression in undergraduate medical students in different semesters.* GMS J Med Educ. 2022;39(1):Doc11. DOI: 10.3205/zma001532

Question 4: Fluid intake (*n* = 301)

	Correct answer	Intuitive concept	Both are incorrect	Uncertainty
**Semester 2**	4,5 %	59,6 %	6,7%	29,2 %
**Semester 6**	10,3 %	61,2 %	9,5 %	19,0 %
**Final year**	7,3 %	71,9 %	13,5 %	7,3 %
**Total**	7,6 %	64,1 %	10,0 %	18,3 %

Question 5: PTT (*n* = 263)

	Correct answer	Intuitive concept	Both are incorrect	Uncertainty
**Semester 2**	3,9 %	47,4 %	7,9 %	40,8 %
**Semester 6**	7,8 %	71,6 %	2,9 %	17,7 %
**Final year**	9,4 %	65,9 %	14,1 %	10,6%
**Total**	7,2 %	62,8 %	8,0 %	22,0 %

Question 6: Antibiotic (*n* = 272)

	Correct answer	Intuitive concept	Both are incorrect	Uncertainty
**Semester 2**	10,1 %	34,2 %	30,4 %	25,3 %
**Semester 6**	22,9 %	20,9 %	44,8 %	11,4 %
**Final year**	43,2 %	6,8 %	47,7 %	2,3 %
**Total**	25,8 %	20,2 %	41,5 %	12,5 %

Question 7: Iron (*n* = 269)

	Correct answer	Intuitive concept	Both are incorrect	Uncertainty
**Semester 2**	55,7 %	12,7 %	11,4 %	20,2 %
**Semester 6**	38,8 %	21,4 %	15,5 %	24,3 %
**Final year**	50,6 %	14,9 %	16,1 %	18,4 %
**Total**	47,6 %	16,7 %	14,5 %	21,2 %

Question 8: Fever (*n* = 287)

	Correct answer	Intuitive concept	Both are incorrect	Uncertainty
**Semester 2**	15,1 %	62,8 %	9,3 %	12,8 %
**Semester 6**	17,1 %	49,6 %	19,8 %	13,5 %
**Final year**	18,9 %	47,8 %	31,1 %	2,2 %
**Total**	17,0 %	53,0 %	20,2 %	9,8 %

Question 9: Febrile convulsion (*n* = 259)

	Correct answer	Intuitive concept	Both are incorrect	Uncertainty
**Semester 2**	18,2 %	33,8 %	6,5 %	41,5 %
**Semester 6**	15,3 %	48,0 %	3,1 %	33,7%
**Final year**	32,1 %	50,0 %	8,3 %	9,6 %
**Total**	21,6 %	44,4 %	5,8 %	28,2 %

Question 10: Disease duration (*n* = 253)

	Correct answer	Intuitive concept	Both are incorrect	Uncertainty
**Semester 2**	38,2 %	40,7 %	7,9 %	13,2 %
**Semester 6**	63,5 %	24,0 %	1,0 %	11,5 %
**Final year**	79,0 %	17,3 %	3,7 %	0,0 %
**Total**	60,9 %	26,9 %	3,9 %	8,3 %

Question 11: Heart attack (*n* = 230)

	Correct answer	Intuitive concept	Both are incorrect	Uncertainty
**Semester 2**	63,3 %	30,9 %	2,9 %	2,9 %
**Semester 6**	86,7 %	10,0 %	0,0 %	3,3 %
**Final year**	91,7 %	5,5 %	1,4 %	1,4 %
**Total**	81,3 %	14,8 %	1,3 %	2,6 %

Question 12: Pacemaker (*n* = 229)

	Correct answer	Intuitive concept	Both are incorrect	Uncertainty
**Semester 2**	28,4 %	41,8 %	5,9 %	23,9 %
**Semester 6**	40,0 %	37,8 %	8,9 %	13,3 %
**Final year**	54,2 %	20,8 %	23,6 %	1,4 %
**Total**	41,0 %	33,6 %	12,7 %	12,7 %

Question 13: Dehydration (*n* = 248)

	Correct answer	Intuitive concept	Both are incorrect	Uncertainty
**Semester 2**	27,4 %	38,4 %	6,8 %	27,4 %
**Semester 6**	25,5 %	38,3 %	11,7 %	24,5 %
**Final year**	50.6 %	21,0 %	22,2 %	6,2 %
**Total**	34,3 %	32,7 %	13,6 %	19,4 %

Question 14: Diuretic (*n* = 244)

	Correct answer	Intuitive concept	Both are incorrect	Uncertainty
**Semester 2**	7,2 %	10,1 %	1,5 %	81,2 %
**Semester 6**	44,0 %	18,7 %	1,1 %	36,2 %
**Final year**	67,1 %	14,5 %	6,6 %	11,8 %
**Total**	40,7 %	14,8 %	3,0 %	41,5 %

Question 15: GFR (*n* = 233)

	Correct answer	Intuitive concept	Both are incorrect	Uncertainty
**Semester 2**	20,6 %	33,8 %	5,9 %	39,7 %
**Semester 6**	26,7 %	42,2 %	2,2 %	28,9 %
**Final year**	46,7 %	41,3 %	8,0 %	4,0 %
**Total**	31,3 %	39,6 %	5,3 %	24,0 %

Question 16: Sodium deficiency (*n* = 261)

	Correct answer	Intuitive concept	Both are incorrect	Uncertainty
**Semester 2**	13,0 %	50,7 %	7,8 %	28,5 %
**Semester 6**	30,7 %	43,6 %	10,9 %	14,8 %
**Final year**	26,5 %	38,6 %	26,5 %	8,4 %
**Total**	24,1 %	44,1 %	14,9 %	16,9 %

Question 17: Smoking (*n* = 281)

	Correct answer	Intuitive concept	Both are incorrect	Uncertainty
**Semester 2**	4,7 %	82,3 %	0,0 %	13,0 %
**Semester 6**	31,8 %	55,1 %	3,7 %	9,4 %
**Final year**	40,4 %	47,2 %	7,9 %	4,5 %
**Total**	26,3 %	60,8 %	4,0 %	8,9 %
